# Proteasome inhibitor-induced autophagy in PC12 cells overexpressing A53T mutant α-synuclein

**DOI:** 10.3892/mmr.2014.3011

**Published:** 2014-11-27

**Authors:** DANMEI LAN, WENZHAO WANG, JIANHUA ZHUANG, ZHONGXIN ZHAO

**Affiliations:** Department of Neurology, Shanghai Changzheng Hospital, Second Military Medical University, Shanghai 200003, P.R. China

**Keywords:** proteasome inhibitor, MG132, autophagy, α-synuclein, Parkinson’s disease

## Abstract

The aim of the present study was to examine the effects of proteasome inhibitor (PI)-induced autophagy on PC12 cells overexpressing A53T mutant α-synuclein (α-syn) by detecting alterations in the levels of microtubule-associated protein 1A/1B light chain (LC3)^+^ autophagosomes and the lysotracker-positive autolysosomes using immunofluorescence, the expression of LC3-II using western blot analysis and the morphology of PC12 cells using transmission electron microscopy. It was found that the addition of MG132 (500 nmol/l) significantly increased the number of autophagosomes and autolysosomes and upregulated the expression of LC3-II. The autophagy inhibitor 3-methyladenine (3-MA) completely inhibited the autophagy induced by MG132 (500 nmol/l). The autophagy enhancer trehalose significantly increased the number of autophagosomes and autolysosomes and improved the protein level of LC3-II induced by MG132. To examine the effect of PI-induced autophagy on the degradation of A53T mutant α-syn, the expression of α-syn was detected by western blot analysis. It was revealed that MG132 increased the expression of A53T α-syn and trehalose counteracted the increase of A53T α-syn induced by MG132. Combined inhibition of 3-MA and PI significantly increased the accumulation of A53T α-syn as compared with treatment using either single agent. In addition, combination of MG132 (500 nmol/l) with trehalose (50 mmol/l) or 3-MA (2 mmol/l) markedly decreased the cell viability as compared with treatment using either single agent individually as demonstrated using a 3-(4,5-dimethylthiazol-2-yl)-2,5-diphenyltetrazolium bromide assay. These results suggest that the PI, MG132, could induce autophagy in PC12 cells overexpressing A53T mutant α-syn and this autophagy could be completely inhibited by 3-MA, indicating that PI-induced autophagy is mediated by the upregulation of the macroautophagy class III PI3K pathway. PI-induced autophagy may act as a compensatory degradation system for degradation of A53T α-syn when the ubiquitin-proteasome system is impaired. Autophagy activation may directly contribute to the survival of PC12 cells treated with proteasome inhibitors. The present study may assist in illuminating the association between PI and autophagy in the pathogenesis of Parkinson’s disease.

## Introduction

Parkinson’s disease (PD) is the second most common progressive neurodegenerative disorder following Alzheimer’s disease, caused by the relatively selective loss of dopaminergic neurons in the substantia nigra. The pathogenic hallmark of PD is the accumulation and aggregation of α-synuclein (α-syn) in susceptible neurons. α-syn is reported to have a central role in PD. It is known that A53T and A30P, two missense mutations in α-syn, cause early-onset autosomal dominant PD (1,2) and transgenic mice with A53T mutant α-syn develop a PD phenotype with Lewy body-like pathology and locomotor impairment (3,4).

α-syn is degraded by the ubiquitin-proteasome system (UPS) and autophagy-lysosome pathways (ALP) (5). Impairment to the UPS and primary non-lysosomal protein degradation pathways have been implicated in PD (6). Failure of the UPS to clear unwanted α-syn, eventually leading to the accumulation and aggregation of α-syn, clearly has a major role in the molecular pathogenesis of sporadic and familial PD (6–8). Several loss-of-function studies on the UPS have provided compelling evidence that UPS impairment is sufficient to cause neural proteinopathy (9–11). Another pathway relevant to α-syn clearance is autophagy, a lysosome-mediated degradative pathway, which mediates the bulk degradation of cytoplasmic proteins or organelles in the lytic compartment. Autophagy involves the formation of double-membrane structures, termed autophagosomes, which fuse with primary lysosomes to become an autophagolysosome. As a result, the contents of the autophagolysosomes are degraded by either disposing or recycling back to cells. Autophagy is regulated by a group of ATG genes. It has been reported that mice that specifically lacked Atg7 in the central nervous system exhibited behavioral defects, massive neuronal loss in the cerebral and cerebellar cortices and accumulation of polyubiquitinated proteins in autophagy-deficient neurons as inclusion bodies (12). Therefore, the impairment to autophagy is implicated in the pathogenesis of neurodegenerative disorders that involve ubiquitin-containing inclusion bodies (13,14). Associated with PD, the A53T mutation of α-syn that readily forms aggregates may be more dependent on autophagy compared with the wild-type protein or A30P mutation (15).

The UPS and ALP have been viewed as independent degradation systems. However, several studies have suggested that they are mechanistically linked (12,16). For example, accumulation of ubiquitin-positive aggregates was observed in Atg7-deficient hepatocytes and neurons and autophagy was induced in response to proteasome inhibition in certain cancer cells in *Drosophila melanogaster* (17–20). In addition, a study in living mouse cortex neurons suggested that the UPS and ALP may be functionally connected such that impairment to either one could upregulate the other (21). However, these mechanisms remain to be clarified and confirmed in the pathogenesis of PD. A PC12 cell line has been created that stably overexpresses A53T mutant α-syn, which is considered an ideal alternative to dopaminergic neurons for PD research. The association between the UPS and ALP in PC12 cells overexpressing A53T mutant α-syn remains to be elucidated. In the present study, this cell line was treated with the proteasome inhibitor (PI) MG132 to see whether it could induce autophagy. This was in order to determine the relevant effects on the degradation of α-syn and survival of PC12 cells and an attempt to gain insights into the mechanism and effect of PI-induced autophagy in the degradation of α-syn associated with the pathogenesis of PD.

## Materials and methods

### Drugs

MG-132, trehalose and 3-methyladenine (3-MA), which were all purchased from Sigma (St. Louis, MO, USA), were dissolved in 100% dimethyl sulfoxide (Sigma) and diluted with Dulbecco’s modified Eagle’s medium (DMEM; Gibco-BRL, Carlsbad, CA, USA) to the desired concentration, with a final dimethyl sulfoxide concentration of 0.1% for *in vitro* study. Trehalose was diluted to 1 mol/l with DMEM. 3-MA was dissolved in dimethylformamide (DMF; Sigma) and diluted with DMEM to the desired concentration, with a final DMF concentration of 0.2% for *in vitro* study. This study was approved by the Ethics Committee of Changzheng Hospital (Shanghai, China).

### Cell culture

A rat PC12 cell line overexpressing human A53T mutant α-syn was constructed using a pEGFP-SNCA-A53T recombinant plasmid (kindly provided by Dr Stephanie Cobb, Mayo Clinic, FL, USA) and the lentiviral gene transfer method. Transfected PC12 cells were further screened with 5 μmol/l blasticidin (Invitrogen Life Technologies, Carlsbad, CA, USA) and obtained using a limiting dilution assay. Stably transfected PC12 cells were cultured in DMEM supplemented with 10% (v/v) heat-inactivated horse serum (Gibco-BRL), 5% (v/v) fetal bovine serum (Gibco-BRL) and blasticidin (5 μmol/l). Cells were cultured at 37°C in humidified air with 5% CO_2_. All experiments were performed 24–48 h after cell seeding.

### Experimental cell treatment

To investigate the effect of an autophagy enhancer or inhibitor on MG132-induced autophagy, the macroautophagy inhibitor 3-MA was applied at a concentration of 2 mmol/l 3 h prior to treatment with MG132 and mammalian target of rapamycin (mTOR)-independent autophagy enhancer trehalose was applied simultaneously with MG132. The effect of MG132 (500 nmol/l) on PC12 cells overexpressing A53T α-syn was evaluated after 24 h incubation. PC12 cells that overexpressed A53T α-syn with solely 3-MA or trehalose for 24 h were used as the control.

### Western blot analysis for microtubule-associated protein 1A/1B light chain (LC3) and α-syn

Total cell lysates of the treated PC12 cells were prepared in ice-cold extraction buffer consisting of 20 mM Tris-HCl (pH 7.4), 10 mmol/l potassium acetate (AcK), 1 mmol/l dithiothreitol, 0.25% NP-40, 1 mmol/l EDTA, 2 mmol/l ethylene glycol tetraacetic acid, 1 mmol/l phenylmethylsulfonyl fluoride and a protease inhibitor cocktail (Sigma), containing 104 mM 4-(2-Aminoethyl)benzenesulfonyl fluoride hydrochloride, 80 μM aprotinin, 4 mM bestatin, 1.4 mM E-64, 2 mM leupeptin and 1.5 mM pepstatin A. The samples were homogenized and centrifuged at 20,000 × g for 10 min at 4°C and then the protein content was determined by the BCA protein assay kit (Pierce Biotechnology, Inc., Rockford, IL, USA). The total quantity of protein (50 μg) was electrophoresed on a 12% SDS-PAGE, transferred to polyvinylidene difluoride, blocked and probed overnight at 4°C with the following primary antibodies: Anti-LC3 antibody (rabbit anti-rat; 1:1,000; Abcam, Cambridge, MA, USA), anti-α-syn antibody (mouse anti-rat; 1:1,000; Sigma) and anti-β-actin antibody (mouse anti-rat; 1:5,000; Sigma). The samples were then incubated with the appropriate secondary antibodies and developed with enhanced chemiluminescence. The blots were quantitated by computer-assisted image analysis software(Quantity One 4.62; Bio-Rad Laboratories, Inc., Hercules, CA, USA).

### Immunofluorescence of autophagosomes and autolysosomes

Cells were seeded on polylysine-coated glass slides (Sigma) in 6-well plates, treated with the experimental drugs, incubated as necessary, stained with 70 ng/ml LysoTracker^®^ Red DND-99 (Invitrogen Life Technologies) for 30 min, washed with phosphate-buffered saline (PBS) at pH 7.2, fixed in 4% paraformaldehyde for 30 min, washed with PBS and permeabilized with 0.1% Triton-X-100 (Sigma) for 15 min, followed by overnight incubation with the LC3 primary antibody (1:1,000; MBL, Nagoya, Japan) and incubation with a secondary antibody (1:100; Kangcheng Biotech, Shanghai, China). A laser-scanning microscope (Olympus BX60; Olympus, Tokyo, Japan) was used to capture images.

### Transmission electron microscopy (TEM)

The presence of autophagic vacuoles in TEM is the gold standard for detecting autophagy. The treated PC12 cells were harvested using 0.25% trypsin, washed with PBS (pH 7.2), collected by centrifugation for 10 min at 440 × g, fixed in ice-cold 2.5% glutaraldehyde in PBS, rinsed with PBS, post-fixed in 1% osmium tetroxide with 0.1% potassium ferricyanide, dehydrated through a graded series of ethanol (30–90%), embedded in Epon (Energy Beam Sciences, Agawam, MA, USA) and sliced into 50–60 nm sections with an LKB-I ultramicrotome (LKB, Bromma, Sweden). The sections were stained with 3% uranyl acetate and lead citrate and finally observed by TEM (Philips CM-120; Philips, Amsterdam, Holland).

### Cell viability assay

Cell viability was measured using a 3-(4,5-dimethylthiazol-2-yl)-2,5-diphenyltetrazolium bromide (MTT) assay. Cells were plated at a density of 2×10^4^ cells per well in 96-well plates. Following treatment with 500 nmol/l MG132 for 24 h, MTT solution (5 mg/ml) was added to each well and the plates were incubated for another 4 h. Subsequently, 150 *μ*l dimethyl sulfoxide was added to each well to resuspend and dissolve the MTT metabolic product. Finally, the optical density was read at 570 nm, with the subtraction of background at 670 nm using a Multiskan MK3 microplate reader (Thermo Electron Corporation, Marietta, OH, USA).

### Statistical analysis

All experiments were repeated at least three times and the data are expressed as the mean ± standard error of the mean. Differences between groups were analyzed by one-way analysis of variance or Student’s t-test using SPSS 12.0 software (SPSS, Inc., Chicago, IL, USA). P<0.05 was considered to indicate a statistically significant difference.

## Results

### Mg132 induces macroautophagy in PC12 cells overexpressing A53T mutant α-syn

**Figure 1:**
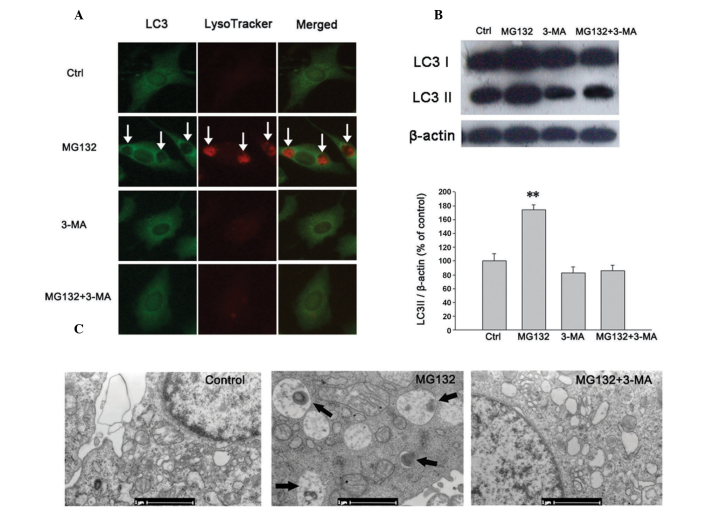
3-MA inhibits MG132-induced macroautophagy and the processing of LC3 in PC12 cells overexpressing A53T α-synuclein. (A) Treatment with 500 nmol/l MG132 was demonstrated to induce the formation of LC3 and Lysotracker RED-positive autolysosomes (white arrows, original magnification, ×400), (B) increase the expression level of LC3-II and (C) affect the morphology of autolysosomes (black arrows) as observed by TEM; these effects could be inhibited completely by 3-MA. (A and C) No evident autophagosomes were detected in 3-MA pre-treated cells, even after 24 h treatment with 500 nmol/l MG132. ^**^P<0.01 vs. control group. 3-MA, 3-methyladenine; LC3, microtubule-associated protein 1A/1B light chain.

**Figure 2:**
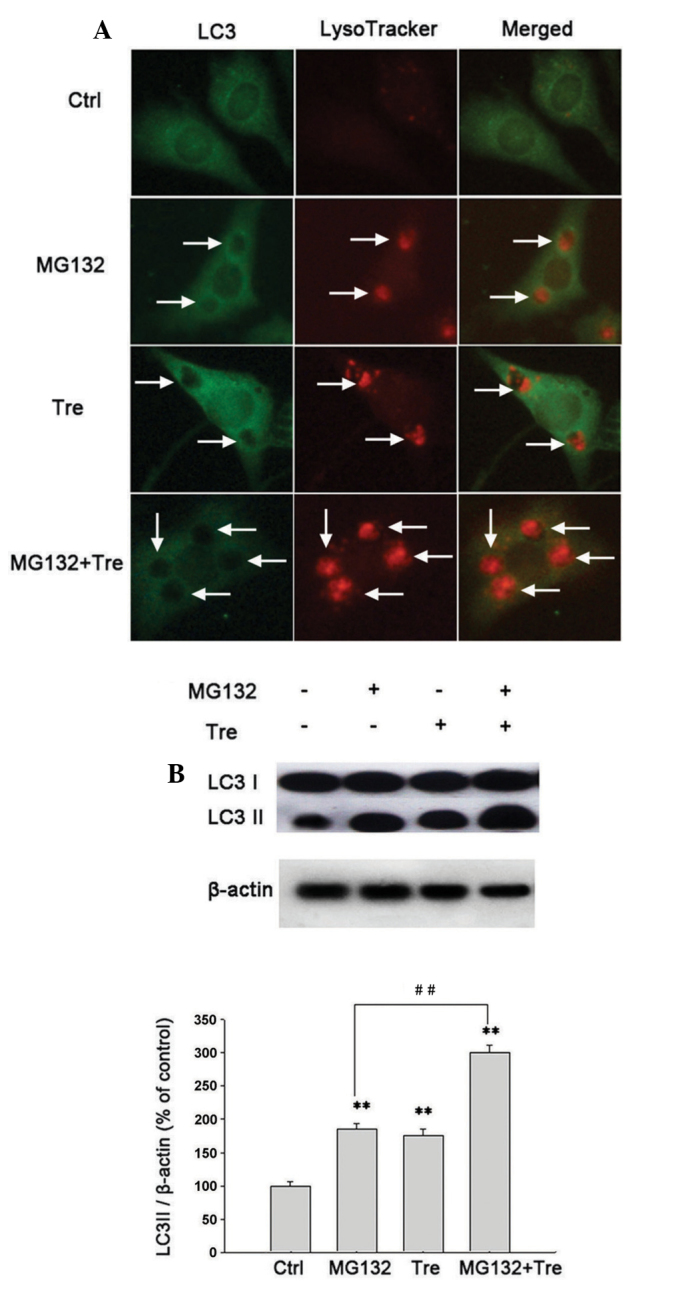
Tre enhances MG132-induced macroautophagy. (A) Inhibition of the ubiquitin-proteasome system significantly activated macroautophagy, and 50 mmol/l Tre further increased the number of LC3^+^ and Lysotracker-positive autolysosomes (white arrows) induced by MG132. Original magnification, ×400. (B) In A53T α-synuclein-overexpressing PC12 cells, 500 nmol/l MG132 significantly increased LC3-II expression (P<0.01). Treatment with 50 mmol/l Tre further upregulated MG132-induced LC3-II expression. ^**^P<0.01 vs. control group, ^##^P<0.01 vs. MG132 group. LC3, microtubule-associated protein 1A/1B light chain; Tre, trehalose.

The induction of autophagy was assessed by detecting an increase in the autophagosomal membrane form of LC3, which is a mammalian homolog of the autophagy-related gene 8 in yeast. It is recruited to the autophagosomal membrane during autophagy, making it a specific marker of autophagy. Since the quantity of LC3 protein, particularly LC3-II, has been previously demonstrated to correlate with the extent of autophagy (22–24), the ratio of LC3II/β-actin was determined in the present study to estimate autophagy. LysoTracker^®^ and LysoSensor™ probes are also commonly used to investigate the acidification of lysosomes and the alterations of lysosomal function or trafficking that occurs in live cells (5,25). To investigate the effect of PIs on autophagy, alterations in LC3^+^ autophagosomes and lysotracker-positive autolysosomes were detected by immunofluorescence. The expression of LC3-II was assayed by western blot analysis and changes in the morphology of PC12 cells were examined by TEM. It was found that in the PC12 cells overexpressing A53T α-syn, MG132 (500 nmol/l) significantly increased the LC3^+^ autophagosome and lysotracker-positive autolysosome levels (Figs. 1A and 2A), upregulated the expression of LC3-II (Figs. 1B and 2B) and promoted the formation of autophagosomes as shown by TEM (Fig. 1C).

### PI-induced autophagy can be inhibited by 3-MA

The class III phosphoinositide 3-kinase (PI3K) inhibitor 3-MA is known to convert LC3-I to LC3-II and is implicated in the modulation of autophagy. To investigate whether the autophagy induced by MG132 in these experiments was mediated by the PI3K pathway, PC12 cells were pretreated with 2 mmol/l 3-MA for 3 h. It was revealed that 3-MA reduced the formation of autophagic vacuoles and the expression of LC3-II protein induced by 500 nmol/l MG132, as shown by immunofluorescence (Fig. 1A), western blot analysis (Fig. 1B) and TEM (Fig. 1C). In summary, it was demonstrated that the presence of MG132-activated macroautophagy in the PI3K pathway can be inhibited by 3-MA.

### Effects of mTOR-independent autophagy enhancer trehalose on PI-induced autophagy

Trehalose is an mTOR-independent autophagy enhancer and can induce autophagy and promote the clearance of A53T α-syn (26). As a positive control, trehalose increased LC3^−^ and Lysotracker RED-positive autolysosome levels following using lysotracker and LC3 staining (Fig. 2A). It also enhanced the expression of LC3-II as shown by western blot analysis (Fig. 2B). In addition, trehalose (50 mmol/l) markedly increased the number of LC3^+^ autophagosomes and lysotracker-positive autolysosomes (Fig. 2A) and the expression of LC3-II induced by MG132 (Fig. 2B).

### Effects of autophagy enhancers or inhibitors on MG132-induced α-syn accumulation

**Figure 3:**
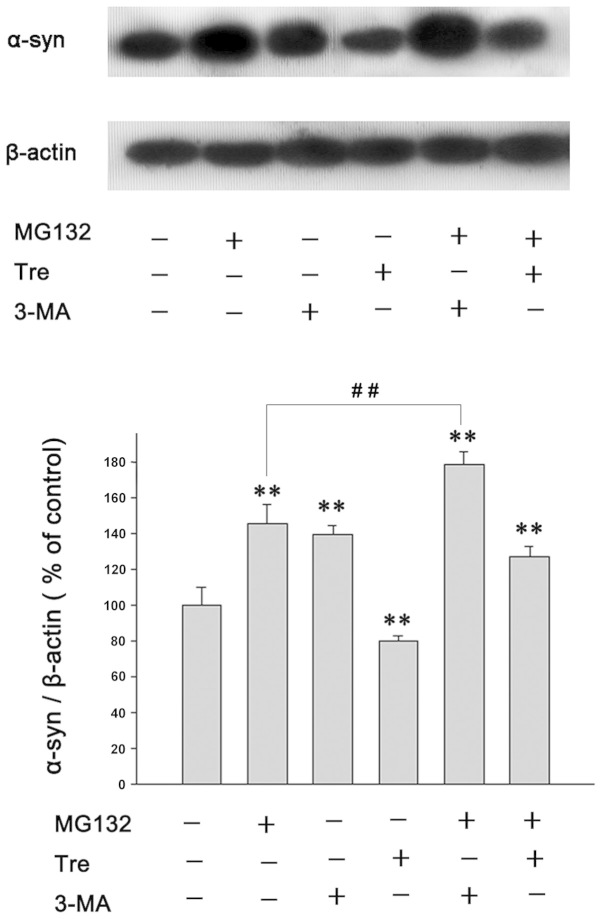
Effects of autophagy enhancers or inhibitors on MG132-induced accumulation of α-synuclein. The A53T α-syn-overexpressing PC12 cells were treated with MG132 (500 nmol/l), Tre (50 mmol/l, tre), MG132 (500 nmol/l) and Tre (50 mmol/l), 3-MA (2 mmol/l), MG132 (500 nmol/l) and 3-MA (2 mmol/l). The expression of α-syn was analyzed by immunoblotting. ^**^P<0.01 vs. control group, ^##^P<0.01 vs. MG132 group. α-syn, α-synuclein; Tre, trehalose; 3-MA, 3-methyladenine.

α-syn can be degraded by the UPS and ALP. To identify the effects of autophagy inducers or inhibitors on A53T α-syn clearance in PC12 cells treated with the PI, A53T α-syn-overexpressing PC12 cells were treated with MG132, with or without trehalose or 3-MA, for 24 h. The results were as follows: MG132 (500 nmol/l) increased the level of A53T α-syn by inhibiting its degradation (Fig. 3) and trehalose (50 mmol/l) attenuated the increase of A53T α-syn induced by MG132 (Fig. 3). Additionally, pretreatment with 3-MA (2 mmol/l) clearly increased the accumulation of A53T α-syn induced by MG132 (Fig. 3), a phenomenon that was similarly observed in previous experiments (27). However, although the inhibition of proteasomes or autophagy, or both, significantly increased the accumulation of A53T α-syn in the present study, immunofluorescence did not detect the inclusion bodies containing aggregated α-syn in PC12 cells (data not shown).

### Effects of autophagy enhancers or inhibitors on PI-induced cell death

**Figure 4:**
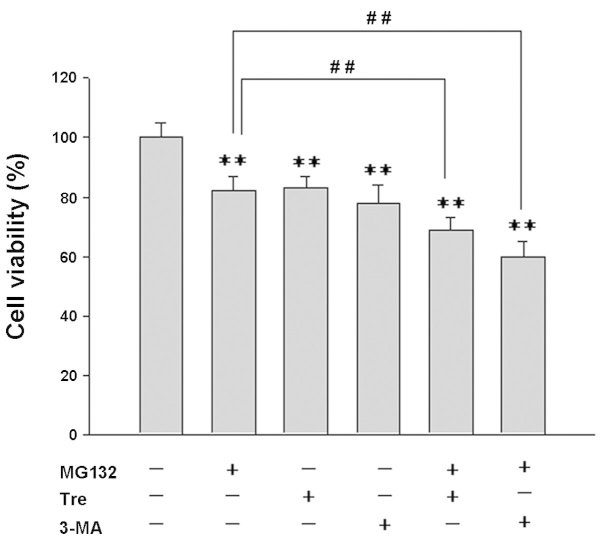
Effects of autophagy enhancers or inhibitors on proteasome inhibitor-induced cell death. The cell viability was measured using a 3-(4,5-dimethylthiazol-2-yl)-2,5-diphenyltetrazolium bromide assay and the results are expressed as the percentage of control. A combination of MG132 (500 nmol/l) with Tre (50 mmol/l) or 3-MA (2 mmol/l) markedly decreased the cell viability compared with treatment using either single agent. ^**^P<0.01 vs. control group, ^##^P<0.01 vs. MG132 group. 3-MA, 3-methyladenine; Tre, trehalose.

To examine the role of PI-induced autophagy in cell survival in A53T α-syn-overexpressing PC12 cells, an MTT assay was used to assess the effect of a combined autophagy enhancer/inhibitor with MG132 on cell viability (Fig. 4). It was found that the combination of MG132 (500 nmol/l) with trehalose (50 mmol/l) or 3-MA (2 mmol/l) markedly decreased the cell viability compared with treatment using either single agent (Fig. 4).

## Discussion

In the present study, the effects of the PI MG132 on PC12 cells overexpressing A53T mutant α-syn, an ideal alternative to dopaminergic neurons, were examined and it was found that MG132 significantly increased the number of autophagosomes and autolysosomes. MG132 also upregulated the expression of LC3-II and promoted the formation of autophagosomes as shown by TEM, thus confirming the presence of PI-induced autophagy in PC12 cells overexpressing A53T mutant α-syn. When the UPS was impaired, it was identified that autophagy operated via a compensatory degradation system. In addition, PI-induced autophagy could be completely inhibited by the selective class III PI3K inhibitor 3-MA, indicating that PI-induced autophagy is mediated by the upregulation of the macroautophagy class III PI3K pathway. Trehalose, an autophagy enhancer, not only induced autophagy in PC12 cells overexpressing A53T mutant α-syn (27), but also significantly improved autophagy induced by MG132. These results indicate that a combination of PI and other autophagy enhancers could produce a synergistic effect in inducing autophagy.

It was also revealed that MG132 increased the expression of A53T α-syn and trehalose counteracted the increase of A53T α-syn induced by MG132. These results are consistent with the previous finding that trehalose accelerated the clearance of mutant α-syn by enhancing autophagy (26). Combined inhibition of autophagy and the proteasome significantly increased the accumulation of A53T α-syn compared with treatment using either single agent. These results suggest that A53T α-syn could be degraded by the UPS and ALP and that PI-induced autophagy may act as a compensatory degradation system for A53T α-syn degradation when the UPS is impaired.

Although treatment with MG132 and/or 3-MA inhibited A53T α-syn degradation and significantly increased its accumulation, immunofluorescence failed to detect inclusion bodies containing aggregated α-syn in PC12 cells in the present study, which may suggest that treatment with PIs is not a reliable model for replicating all the neuropathological abnormalities of PD.

Ample evidence supports the hypothesis that autophagy is a survival pathway required for cellular viability (28). In the present study, it was also observed that there is increased cell death in PC12 cells following treatment with 3-MA alone. In addition, the effect of PI-induced autophagy on the survival rate of PC12 cells overexpressing A53T α-syn was examined and it was revealed that pre-treatment with 3-MA potentiated PI-induced cell death *in vitro*, as determined by measuring cell viability with an MTT assay. These findings indicate that autophagy activation may directly contribute to the survival of PC12 cells treated with proteasome inhibitors.

Several lines of evidence have suggested that autophagy enhancers can promote the clearance of aggregate-prone proteins and decrease PI-induced cell death (29,30). However, in the present study combined treatment with MG132 and trehalose resulted in a marked decrease in cell viability in A53T α-syn-overexpressing PC12 cells compared with treatment by MG132 alone. It was also supported by a previous study, which may be explained by the change of osmolality and overactivated autophagy induced by trehalose (27).

In conclusion, in the present study it has been demonstrated that PI can induce macroautophagy in PC12 cells overexpressing A53T α-syn. The UPS and ALP are two reciprocally regulated, not strictly parallel degradation systems that are important in the cellular mechanisms of A53T α-syn degradation. PI-induced autophagy can be completely inhibited by the autophagy inhibitor 3-MA, suggesting that such action depends on class III PI3K. The combination of PI and trehalose synergistically promotes autophagy. Trehalose can offset IP-induced accumulation of A53T α-syn. The inhibition of autophagy can significantly increase the accumulation of A53T α-syn and proteasome inhibitor-induced cell death, indicating that PI-induced autophagy may have a cytoprotective role in the degradation of A53T α-syn and in the survival of PC12 cells overexpressing A53T α-syn. These results may prove useful in illuminating the mechanism of PI-induced autophagy in the pathogenesis of PD.
